# Cost-Effective Marine Protection - A Pragmatic Approach

**DOI:** 10.1371/journal.pone.0147085

**Published:** 2016-01-11

**Authors:** Soile Oinonen, Kari Hyytiäinen, Lassi Ahlvik, Maria Laamanen, Virpi Lehtoranta, Joona Salojärvi, Jarno Virtanen

**Affiliations:** 1 Finnish Environment Institute, Helsinki, Finland; 2 University of Helsinki, Helsinki, Finland; 3 Natural Resources Institute Finland, Helsinki, Finland; 4 Ministry of the Environment, Helsinki, Finland; US Army Engineer Research and Development Center, UNITED STATES

## Abstract

This paper puts forward a framework for probabilistic and holistic cost-effectiveness analysis to provide support in selecting the least-cost set of measures to reach a multidimensional environmental objective. Following the principles of ecosystem-based management, the framework includes a flexible methodology for deriving and populating criteria for effectiveness and costs and analyzing complex ecological-economic trade-offs under uncertainty. The framework is applied in the development of the Finnish Programme of Measures (PoM) for reaching the targets of the EU Marine Strategy Framework Directive (MSFD). The numerical results demonstrate that substantial cost savings can be realized from careful consideration of the costs and multiple effects of management measures. If adopted, the proposed PoM would yield improvements in the state of the Baltic Sea, but the overall objective of the MSFD would not be reached by the target year of 2020; for various environmental and administrative reasons, it would take longer for most measures to take full effect.

## Introduction

Deterioration of marine environments due to various anthropogenic pressures [1–3] has become a global concern. In Europe, the EU Marine Strategy Framework Directive (MSFD) [4] embodies the key environmental policy for addressing this challenge, setting as it does the ambitious aim of achieving and maintaining Good Environmental Status (GES) of the European marine waters by 2020. These areas include the Black, Mediterranean, North and Baltic Seas. The MSFD establishes an ambitious international policy, in terms of not only its environmental objective and the speed with which it is to be reached, but also the wide scope and holistic nature of the analyses required in its application. The Directive explicitly requires the member states to assess the present state of the sea in question and develop a national Programme of Measures (PoM) designed to narrow and, eventually, to close the gap between the current and desired state of the sea. Moreover, the member states must show that the chosen PoM is cost-effective.

The implementation of the MSFD calls for adoption of a form of ecosystem-based management (EBM) in which marine protection and delivery of the ecosystem goods and services are taken care of concurrently [5, 6]. Thus, trade-offs are unavoidable and decision support tools are needed that provide a holistic view on the consequences of alternative management measures and related uncertainties. The development of such tools is complicated by the fact that the analyses are needed ex ante; that is, the ecological response functions and the costs and benefits of the measures must be determined before implementation. This in turn requires multi-disciplinary data or modelling results; moreover, data are often lacking and must be extrapolated, or elicited from experts. [7, 8]. EBM in the present case poses a transdisciplinary challenge that to date may well have confined the development of tools to the conceptual level. There is a growing literature illustrating that the Drivers-Pressures-State-Impact-Response (DPSIR) concept and its various modifications help in educating managers about the complexity of marine ecosystems and their links to human wellbeing [9–12]. What is lacking is a tool that helps decision makers to select a cost-effective set of measures to meet a given multidimensional environmental objective. The present paper provides a framework for probabilistic and holistic cost-effectiveness analysis (CEA) that makes it possible to consider the relative performance of alternative management measures, to rank them and to develop the least-cost combinations of measures to achieve GES. The framework is applied in choosing the optimal PoM for Finland.

Earlier experiences of economic analyses applied in implementing the Water Framework Directive (WFD) [13]show that once reliable estimates of the effectiveness and costs of measures are available, a CEA is straightforward [14, 15]. The MSFD poses particular difficulties for analyses due to the multidimensional description of the environmental objective involved and the substantial data needed for them. The Directive applies a holistic functional approach that takes into account the structure, function and processes of marine ecosystems [16]. GES is defined using 11 qualitative descriptors (Table 1). The overall GES assessment is further complicated by the fact that the descriptors are hierarchical and interlinked such that changes in some descriptors may have an impact on others. For example increased eutrophication (D5) can have undesirable impacts on food web functioning (D4) [17]. Clearly, the effectiveness of candidate PoMs must be defined in these terms, which calls for a multidimensional objective function. The European Commission has issued a decision [18] on the criteria and methodological standards to be used in determining GES of marine waters, but the operationalization of GES—defining the environmental objective in quantitative terms—is largely left to the member states [17]. Moreover, the MSFD does not give any guidance on how to weight the gaps in the attainment of different GES descriptors [19]. Therefore, as has been the case in studies on compliance with the WFD [20], a CEA for the MSFD must be carried out without a quantitative definition of environmental objectives and the related thresholds [21].

**Table 1:** Qualitative descriptors for determining good environmental status (GES) in the MSFD [4].

MSFD descriptor	Short name	Abbreviation
Biological diversity is maintained. The quality and occurrence of habitats and the distribution and abundance of species are in line with prevailing physiographic, geographic and climatic conditions.	Biodiversity	D1
Non-indigenous species introduced by human activities are at levels that do not adversely alter the ecosystems	Non-indigenous species	D2
Commercially exploited fish and shellfish	Commercially exploited fish and shellfish	D3
All elements of the marine food webs, to the extent that they are known, occur at normal abundance and diversity and levels capable of ensuring the long-term abundance of the species and the retention of their full reproductive capacity.	Marine food webs	D4
Human-induced eutrophication is minimised, especially adverse effects thereof, such as losses in biodiversity, ecosystem degradation, harmful algae blooms and oxygen deficiency in bottom waters.	Human-induced eutrophication	D5
Sea-floor integrity is at a level that ensures that the structure and functions of the ecosystems are safeguarded and benthic ecosystems, in particular, are not adversely affected.	Sea floor integrity	D6
Permanent alteration of hydrographical conditions does not adversely affect marine ecosystems.	Hydrographical conditions	D7
Concentrations of contaminants are at levels not giving rise to pollution effects.	Concentrations of contaminants	D8
Contaminants in fish and other seafood for human consumption do not exceed levels established by Community legislation or other relevant standards.	Contaminants in fish and other seafood	D9
Properties and quantities of marine litter do not cause harm to the coastal and marine environment	Marine litter	D10
Introduction of energy, including underwater noise, is at levels that do not adversely affect the marine environment.	Energy, including underwater noise	D11

A fundamental feature of a holistic cost-effectiveness analysis is the substantial uncertainty on both costs and effect of different measures on closing the gap. A successful CEA would require access to relevant numerical models. Ideally, the analysis would be carried out using an ecosystem-economic model accounting for the 11 descriptors and their interlinkages, but such models do not exist. As the second-best option, cost-effectiveness could be analyzed by individual management measure and by the GES descriptor it relates to. This was the principal approach applied in corresponding WFD analyses [20]. Unfortunately, for most of the GES descriptors there are no models that could be used to establish a dose-response link between management measures and the objectives set out in each descriptor; what is more, such an approach would not be in line with EBM. This paper seeks to address this shortcoming by shedding light on how to conduct CEA for the implementation of the MSFD and opening research avenues in this area.

A study comparing qualitative and quantitative approaches to CEA for the WFD argues that CEA should meet three core requirements: 1) transparency, 2) pragmatism and 3) usefulness [14]. By transparency the authors of the study mean that the analysis and outcomes are readily understandable by decision makers, and usefulness that they provide true support for the decision-making process. We agree with all three criteria, but redefine pragmatism, which in the study cited meant that the analysis could be carried out by a non-economist. In the present paper, ‘pragmatic’ means that an economically sound analysis can be carried out even under very strict constraints as regards data, knowledge, skills and time for completing the analysis. On balance, our framework can be considered transparent, pragmatic and useful: it includes a methodology for deriving and populating criteria for effectiveness and costs, ranking measures based on their cost-effectiveness and selecting the least-cost combination of measures.

The contribution of the present study is to develop a probabilistic approach for cost-effectiveness analysis of marine protection projects that is in line with the EBM. The framework will be demonstrated through its capacity to rank candidate measures and identify cost-effective combinations of measures that can be incorporated into the final Finnish PoM. In light of the iterative nature of the planning of measures and of the Directive, which follows an adaptive management cycle of six years, the framework has been designed to allow for numerical computations with limited data and limited human resources, and to be flexible enough to be amended during subsequent cycles. The framework is general in that it shows the steps needed to execute a theoretically sound CEA and provides a pragmatic application of such an analysis. Thus the framework is applicable in other contexts, such as water quality or fisheries management, biodiversity conservation or marine spatial planning, where decision support tools are needed to analyze complex ecological-economic trade-offs under uncertainty [8, 22–25].

The rest of the paper is organized as follows. The second section describes the step-by-step CEA framework for implementation of the MSFD as well as an application of the framework–including data collection—for Finland. The third section presents the results, which include a ranking of the measures as well as cost-effective combinations of measures. The last section concludes the study and paves the way for further development of CEA methodology.

## Materials and Methods

### Framework for analyzing and developing cost-effective candidate Programmes of Measures

**Fig 1:**
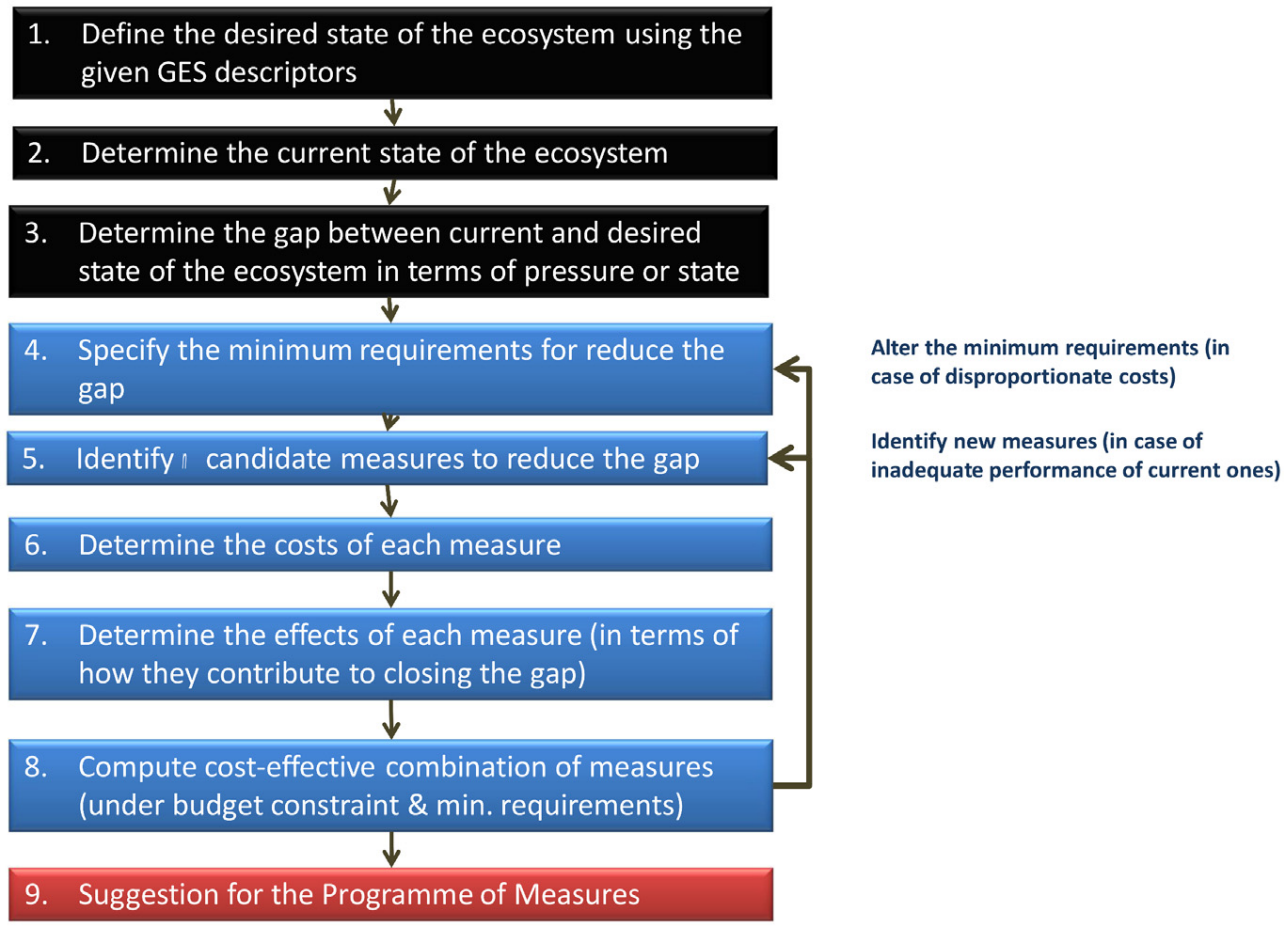
Framework used for developing a national PoM designed to achieve or maintain Good Environmental Status.

Fig 1 shows the step-by-step process of data acquisition and analysis in the preparation of candidate PoMs for public hearing, public discussion or additional consideration. The first three steps include the specification of GES and description of the gap between the present marine status and GES. In general—and especially as a precondition for CEA—GES, the present status of the marine area, and the potential gap between the two should be described quantitatively on an interval or ratio scale and in terms of some well-established and generally accepted criteria. The fourth task is to determine the minimum requirements for the joint effects of measures in closing the gap. These requirements can be specified in terms of some minimum probability that the target will be met or, if achieving GES is unrealistic, some lower, intermediate target. Steps 5–7 involve exploring candidate measures for closing the gap and describing the costs and effectiveness of each measure. The costs and effects can be expressed either as point estimates or as probability distributions. The preferred sources of such information are coupled economic-ecological models or integrated assessment models developed to evaluate the societal and ecological consequences of environmental policies or measures. If suitable tools with adequate scope are not available for simulation, the cost and effect estimates can be based on expert opinions collected in some organized and well-structured manner.

The eighth step is to compute a cost-effective combination of measures that will meet the minimum requirement for GES under an exogenously given budget constraint. The resulting candidate PoM is then analyzed in terms of whether it contributes to closure of the gap in a balanced manner across different GES descriptors and within the budget. Where new measures are identified or the budget constraint changes, steps 4–8 are repeated until a satisfactory PoM is found.

### Developing the Programme of Measures for Finland

In Finland the MSFD has been transposed by updating the Act on Water Resources Management [26], the law that implemented the WFD. This has been supplemented by the Government Decree on organizing the Finnish Marine Strategy planning process [27]. Preparation of the Finnish PoM falls within the remit of the Ministry of the Environment, which coordinates the process and carries out the work in cooperation with the Ministry of Agriculture and Forestry and the Ministry of Transport and Communication. Also collaborating are the regional Centres for Economic Development, Transport and the Environment. In the present case, preparation of the national PoM was led by the Working Group established for the purpose for and carried out in several sub-working groups in 2013–2014. The members of the groups comprised planning and other relevant officials, researchers and NGOs. In total, over sixty people participated in dozens of meetings geared to completing the process.

The sub-working group whose mandate was to conduct the CEA was established at a rather late stage, when steps 1–5 (Fig 1) had already been taken. The conclusion put forward by natural scientists and officials was that there is a gap between the present status of the marine environment and GES for a number of descriptors. In addition, a first screening of the measures proposed for achieving GES, based on technical feasibility and social acceptability, was carried out before the economic analysis. Ultimately, the process identified a total of 31 candidate measures for consideration in the cost-effectiveness analysis.

### Probability distributions for the effects of measures

From the very beginning of its work, the socio-economic working group realized that the potentially applicable ecological-economic models available covered only few of the GES descriptors. Moreover, the models available for non-indigenous species (D2), commercial fisheries (D3) and eutrophication (D5) would require substantial modifications if they were to be applied in the MSFD context [28–32]. This being the case, it was agreed that the cost and effects of the measures would be assessed using expert knowledge and structured interviews.

In our application, effectiveness is defined in terms of a probability distribution describing the likelihood that a particular measure will bridge given proportions of the gap between the present status and the minimum threshold for reaching GES. Note that GES is not a synonym for undisturbed virgin state of marine ecosystem, but rather includes states that tolerate some pollution and consumptive use. The advantages of this approach are that 1) the probability distribution can be flexibly parameterized using data sets, models, expert judgments or a combination of these; 2) probabilistic analysis allows us to study uncertainties related to the success of the candidate PoMs, an important feature called for in the earlier literature [15, 33, 34]; and 3) it has a direct link to the gap analysis preceding the establishment of new measures. To parameterize the effectiveness indicator, we explored and then suggested several alternative approaches to the thematic expert groups tasked with evaluating the effects and costs of the candidate measures. We first attempted to apply a qualitative matrix approach, as described in [35] for example, and then triangular distributions, as applied in [33]. However, the experts did not endorse either of these suggestions. After several unsuccessful and rejected efforts, we proposed an effectiveness indicator in the form of a discrete conditional probability distribution. The experts accepted this definition surprisingly easily. The idea is similar to that used in populating Bayesian Belief Network (BBN) models, which are widely used for decision support in various fields [36–38]. Several successful applications of BBNs can be found in both the natural and the social sciences [23, 24, 39–41]. The costs of the measures were also defined and elicited using a conditional probability distribution.

The particular challenge that MSFD poses for CEA is the large number of GES descriptors. A multidimensional environmental objective such as GES makes data collection a laborious and demanding task. First, it is very likely that several management measures affect more than one descriptor, and any analysis should account for and estimate all these multiple impacts. A second and even more challenging consideration is that measures may have antagonist, additive or synergetic effects on each other [35]. Given these circumstances, an analysis should be able to take into account the joint probability distributions for all combinations of measures—an impracticable task if the number of measures is large. Pragmatism and the cognitive limits of experts called for reasonable assumptions about the interrelationship of measures [14, 35, 42]. Ultimately, we assumed that the impacts of measures are mutually independent.

### Data acquisition through structured interviews

The proposed definitions of the costs and effects of the measures and the assessment method were tested and further developed after a pilot email questionnaire conducted spring 2014. Based on the survey findings, it was decided that the cost and effect data would be acquired using group interviews following the procedure set out in Table 2. Covering all the seven steps in one interview turned out to be too demanding in terms of allocated time and cognitive burden. Ultimately, each interview covered only steps 1–4 in full; steps 5–7 were covered only in part and thus left out of the present analysis. In all, six interviews were conducted in thematic expert working groups that were established earlier in the process of developing candidate PoMs. The interviews eliciting probabilities started with a warm-up exercise in which the group members raised their hands if they thought that a given measure could have the size of effect described by the given state of the probability distribution. The group members had seven votes per measure and thus could vote for a positive probability for each of seven states. The facilitator then drew a uniform distribution over the states that received votes, after which the group discussed the probabilities assigned to each state. The outcome of the vote-tallying exercise was not recorded and was not binding, whereby those states that did not receive any votes could still get a positive probability. The facilitator emphasized that the wider the distribution the higher the uncertainty and this eased the cognitive burden of the experts. The facilitator played an important role in capturing the individual variability in opinions in the probability distributions. If, for example, were two differing views prevailed on the states of the distribution, the facilitator assigned probabilities to these states and the numbers were changed until the group reached a consensus. Table 3 shows the themes taken up by the groups, the number of experts and the number of candidate measures assessed. In total, 41 candidate measures were proposed and their costs and effectiveness were assessed, but 10 were excluded during the process leading to the public hearing; accordingly, the present CEA considers 31 candidate measures (Table 4). The supporting material shows the conditional probability distributions for the costs and effectiveness of each of these measures (S1 and S2 Tables).

**Table 2:** Steps of the group interviews to assess the costs and effects of candidate measures.

Steps of the group interviews to assess the costs and effects of candidate measures
1. Common understanding of the gap with respect to each of the GES descriptors
2. Common understanding of the content and cause-effect mechanism of the candidate measure
3. Assessment of the effectiveness of the candidate measure
4. Assessment of the costs of the candidate measure
5. Assessment of the difficulty of the cost and effectiveness assessment
6. Assessment of the joint effect of the candidate measures
7. Assessment of the cross-effect of the candidate measures

**Table 3:** Cost-effectiveness workshops with thematic experts.

Workshop theme (date)	Number of experts	Number of candidate measures assessed
Eutrophication (18.9.2014)	13	6
Commercial fish stocks (19.9.2014)	6	7
Biodiversity (22.9.2014)	8	10
Marine traffic(2.10.2014)	4	4
Marine litter (6.10.2014)	7	8
Hydrography, underwater noise and toxic substances (7.10.2014)	6	6

**Table 4:** List of candidate measures.

Measure	Description
M1	Reduce food production and consumption impacts on water
M2	Influence agri-environmental compensation mechanism to improve water conservation
M3	Promote the commercialization and deployment of fish feed based on raw materials produced in the Baltic Sea region
M4	Improve habitats of sensitive species living in waters discharging into the sea
M5	Implement nutrient-neutral municipal pilot projects
M6	Study coastal species fisheries management and its efficiency
M7	Implement national strategy for the Baltic Salmon and sea trout
M8	Protect mullet
M9	Incorporate conservation objectives of the marine protected areas into marine spatial plans
M10	Enhance protection of marine conservation areas
M11	Develop programmes of measures for endangered species and habitats
M12	Produce material for education and communication about the state of and pressures on the marine environment
M13	Protect Baltic ringed seal
M14	Conduct impact assessments for small-scale dredging
M15	Decrease oil accident risks in ship to ship operations by tighter regulation in the Finnish waters
M16	Promote NOx Emission Control Areas (NECAs) in the Baltic Sea
M17	Promote LNG as fuel for ships and provide the necessary infrastructure
M18	Promote decisions of the International Maritime Organization to reduce ship underwater noise
M19	Reduce impulsive noise caused by underwater construction
M20	Reduce underwater noise
M21	Reduce use of plastic bags
M22	Increase the efficiency of micro-dust removal from waste water
M23	Influence EU to reduce the use of micro-plastics in cosmetics and hygiene products
M24	Improve off-port waste reception capacity
M25	Improve waste management at waterfront recreational sites
M26	Cooperate with fishermen to reduce marine litter
M27	Reduce and eliminate ghost nets
M28	Reduce litter
M29	Implement measures to improve local flow conditions in the coastal area
M30	Conduct a study of pharmaceutical substances in the Baltic Sea
M31	Explore the meaning of the Kymi river as a source of dioxin in the Baltic Sea

The effectiveness of a candidate measure was defined using a discrete conditional probability distribution (Table 5). The expert groups evaluated each candidate measure against its ability to bridge the gap to be filled for each GES descriptor separately. An effectiveness indicator was calculated as the sum of a linear scoring system. The costs of candidate measures were also elicited using a discrete conditional probability distribution for the total costs of the measure during the period 2016–2022 (Table 6). The experts included both direct and indirect costs in their assessments.

**Table 5:** Effectiveness of a candidate measure as a conditional probability distribution and the related scores.

Class	Description	Score
1	Measure has no impact	0
2	Measure bridges < 12.5% of the gap	0.063
3	Measure bridges 12.5–25% of the gap	0.188
7	Measure bridges 25–50% of the gap	0.375
5	Measure bridges 50–75% of the gap	0.625
6	Measure bridges 75–100% of the gap	0.875
7	Measure achieves GES by 2020	1.000

**Table 6:** Costs of a candidate measure as a conditional probability distribution and related scores.

Class	Description	Score
1	0–0.1 M€	0.05
2	0.1–0.5 M€	0.3
3	0.5–1 M€	0.75
7	1–5 M€	3
5	5–10 M€	7.5
6	10–50 M€	30
7	>50 M€	50

### Ethics statement

According to the guidelines of the Finnish Advisory Board on Research Integrity the nature of this research did not require ethical review. URL: http://www.tenk.fi/en/ethical-review-human-sciences/ethical-review#_ftnref1. The study is based on material collected in working groups appointed by the Finnish Ministry of Environment; therefore consent was not explicitly recorded. Workshops did not include any personal questions and the material underlying this study is the outcome of group discussions. The material (see Supplementary material) was publicly available during the public hearing for the Finnish PoM.

### Problem formulation

There are several alternative ways to formulate the problem of developing economically justified suggestions for candidate PoMs. For example, the choice of measures can be based on maximizing improvement in the environmental state under some exogenously given budget constraint, or minimizing the aggregate cost under some minimum requirement. Multi-objective optimization maybe used in the case of conflicting objectives. The selected formulation may reflect the aims or aspirations of society, the scarcity of resources, the nature and profusion of the objectives involved or the coverage and quality of data available for analysis.

In a general form, the management problem can be defined as one of selecting the combination of candidate measures that maximizes the expected aggregate welfare or utility, *E[U]*, under a particular budget constraint. Societal utility reflects the improved provision of marine ecosystem services to society and the marginal value of these services. Utility is described here as the ability of the focal PoM to close the gap between the current and the desired state of the marine environment:

$$\underset{\left\{x_{i},i=1,\ldots ,n\right\}}{\max}\overset{m}{\underset{j=1}{\sum }}\gamma_{j}E\left[U\left(\overset{n}{\underset{i=1}{\sum }}\delta_{j}f_{ij}x_{i}\right)\right].$$

The probability distributions describing the contribution of *n* = 31 measures to closing the gaps in the case of m = 11 GES descriptors are given by*fij*. The decision variables, *x*_*i ,*_are binary variables, and they denote whether the i^th^ measure becomes part of the PoM or not. The implicit assumption is that the GES descriptors are separable and that the societal utility derived from improvements in the 11 GES descriptors are additive, meaning that the goals are interchangeable and complementary.

Initially, the gap between the present environmental status and GES might differ from descriptor to descriptor. A scaling factor, *δ*_*j*_, denotes the relative severity of the gap in the case of each GES descriptor, and it may be given as a proportion expressing the value of the current state vis-à-vis the value envisaged by the descriptor. Another scaling factor, *γ*_*j*_, denotes the relative weight society gives to meeting the GES descriptors and reflects the marginal value of the ecosystem services provided if the gap is closed.

The joint effect of measures is maximized subject to a budget constraint:

$$E\left[\overset{n}{\underset{i=1}{\sum }}c_{i}x_{i}\right]\leq \overset{´}{c},$$

where *c*_*i*_denotes the probability distribution for the cost of the i^th^ measure and ć denotes the overall budget constraint. In addition, a decision maker may set some minimum requirement ${\overset{´}{f}}_{j}$ for each GES descriptor:

$$E\left[\overset{n}{\underset{i=1}{\sum }}f_{ij}x_{i}\right]\geq \overset{´}{f_{j}}forallj.$$

The expected costs and effects of measures imply a risk-neutral decision maker. However, as the costs and effects of measures are expressed as probability distributions, risk aversion and rankings based on stochastic dominance may also be taken into account.

### Computations

The choice of the optimal combination of measures is a binary optimization problem. Such problems are computationally challenging. There is no way of guaranteeing that the global optimum is reached unless the outcomes of alternative combinations of measures can be exhaustively explored. In this study, the number of alternative candidate PoMs was too high (2^31^) to be comprehensively evaluated and thus a heuristic was employed to search for the optimal solutions and utility possibility frontiers for various budget constraints. The solutions were derived by first ranking alternative measures based on the cost-to-effect ratios of meeting individual GES descriptors and then including the most promising measures in the focal PoM first. For comparison, the outcomes were computed for a large number of randomly selected combinations of measures, as well as for combinations of randomly selected measures and the best candidate measures.

## Results

### Cost-effectiveness ranking of the measures

To start with, we evaluate the costs and effects of individual measures in closing the gap between the initial and desired state of the environment. Table 7 lists all 31 candidate measures and illustrates how each contributes to closing the gap with respect to each of the 11 different GES descriptors. The last column gives the expected overall cost of the measure during six-year management cycle. For each GES descriptor the color codes reveal the four most effective measures relative to the associated cost. The distribution of costs across GES descriptors is assumed to be proportional to the distribution of effects.

**Table 7:** Expected cost and effectiveness of the 31 candidate measures on 11 GES descriptors. The ranking of the four best measures based on cost-to-effect ratio is shown in parenthesis.

	Expected effectiveness of candidate measures on GES descriptors D1-D11(Table 1)											Expected cost
Measure	D1	D2	D3	D4	D5	D6	D7	D8	D9	D10	D11	MEUR
M1	0.013			0.013	0.063 (4)	0.006						2.9
M2	0.038			0.038	0.119 (1)	0.013 (2)						0.7
M3	0.004			0.004	0.038							2.9
M4	0.025			0.013	0.013							7.3
M5	0.002			0.001	0.019 (3)	0.001						0.5
M6			0.006									0.1
M7	0.075		0.144									11.6
M8	0.044											2.3
M9	0.119	0.006 (3)	0.069	0.025	0.003	0.125	0.006 (3)				0.063	21.9
M10	0.200		0.125			0.094 (4)				0.031	0.050 (4)	2.8
M11	0.250	0.025 (2)	0.031	0.019		0.044					0.013 (3)	18.8
M12	0.056 (2)	0.013 (1)				0.050 (1)				0.038 (1)	0.019 (2)	0.2
M13	0.056			0.075								0.9
M14	0.044			0.006	0.006 (2)	0.081 (3)	0.006 (2)					0.7
M15	0.021		0.006	0.013				0.031 (2)	0.006			1.6
M16	0.003		0.003	0.003	0.031							2.6
M17	0.003		0.003	0.003	0.031							50.0
M18	0.031		0.031	0.031							0.075	1.3
M19	0.044 (1)		0.006 (1)	0.050 (1)							0.388 (1)	0.3
M20	0.050		0.000								0.050	0.5
M21	0.031		0.031 (3)	0.038 (4)					0.019 (2)	0.069 (3)		0.3
M22	0.019		0.019	0.025					0.006	0.063		0.8
M23	0.013 (3)		0.013 (2)	0.019 (2)					0.003 (1)	0.050 (2)		0.1
M24	0.006		0.006 (4)	0.013					0.006 (3)	0.031 (4)		0.1
M25	0.038		0.038	0.044					0.025 (4)	0.094		1.1
M26	0.006		0.006	0.013					0.006	0.031		0.4
M27	0.006		0.019	0.013					0.006	0.031		0.4
M28	0.044		0.044	0.050 (3)					0.031	0.100		2.0
M29	0.063 (4)			0.031			0.500 (1)					0.8
M30								0.006 (3)	0.006			0.4
M31								0.003 (1)	0.003			0.1

Table 7 demonstrates that most measures have effects on several GES descriptors. Most measures have a positive effect on descriptors D1 and D4, while only one measure contributes to achieving D7, a case in which the state of the environment is already considered to be GES. The costs also vary considerably: Most measures (17 out of 31) cost less than 1 MEUR, while the most expensive measure costs 50 MEUR.

The preliminary ranking of alternative measures set out in Table 7 provides a first look at the potential and relative performance of each measure in closing the gap for each GES descriptor. However, the choice of the cost-effective PoM calls for calculating the joint effect and total cost of a set of measures and defining the weights society gives to various GES descriptors.

### Joint effects of measures and their aggregate costs

**Fig 2:**
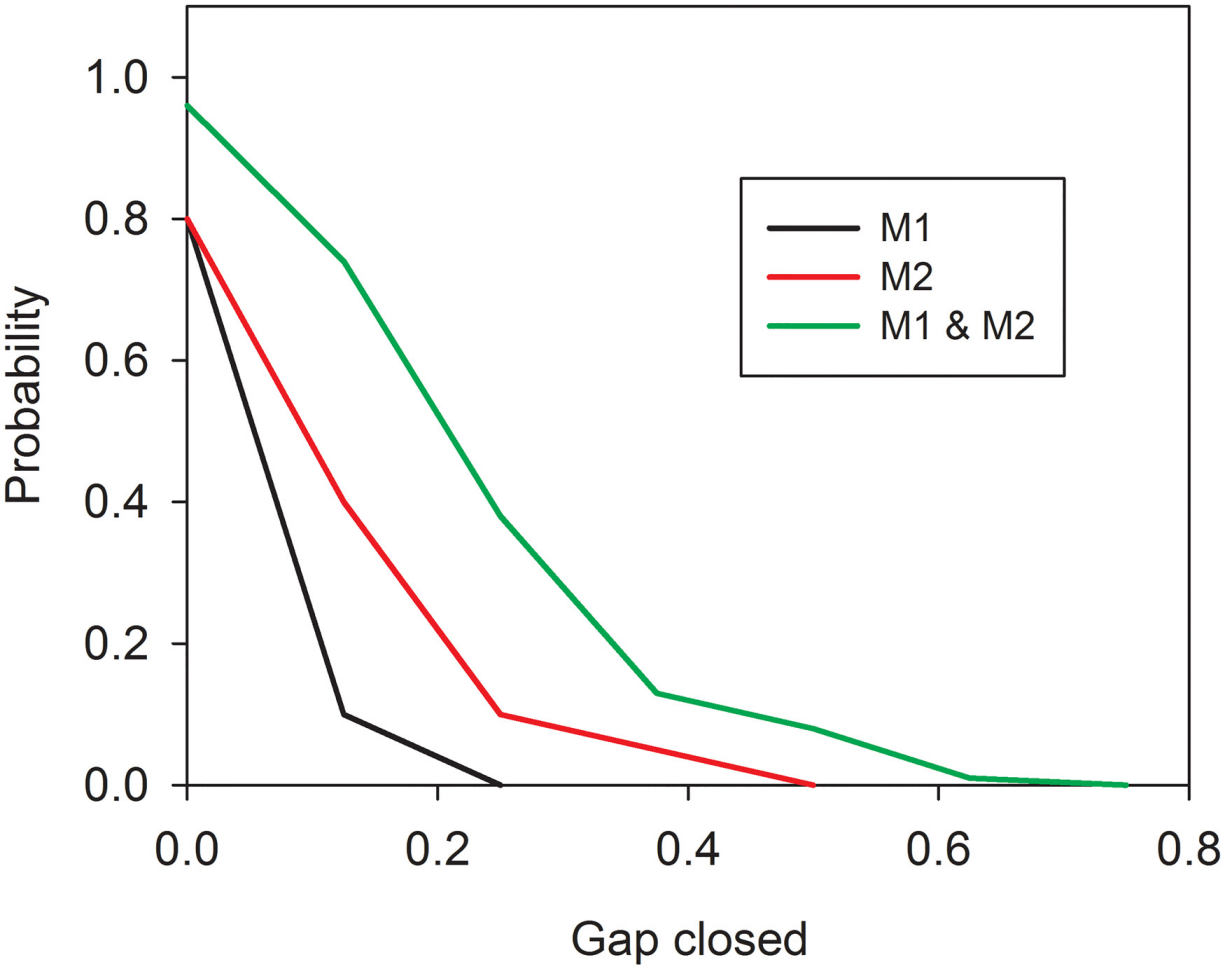
Effectiveness of measures M1 and M2 and their joint contribution to close the gap with respect to descriptor D5.

**Fig 3:**
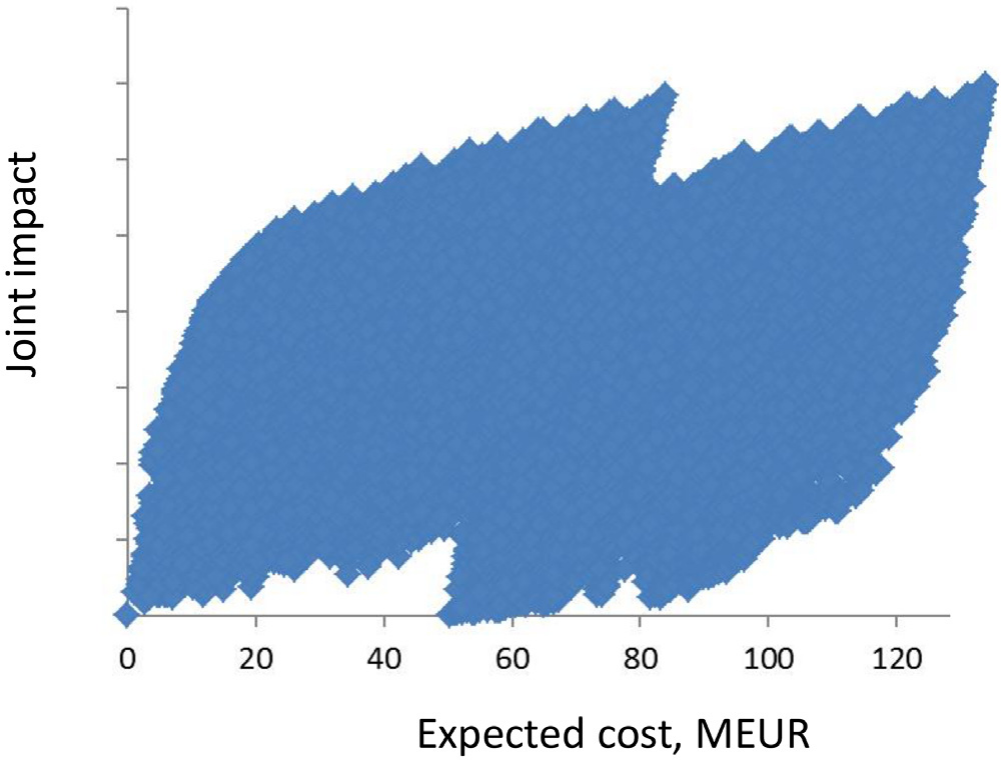
The expected impacts and costs for a large number of alternative combinations of candidate measures. The impact is determined here as the joint impact on the 1^st^, 4^th^, 5^th^, 8^th^ and 9^th^ descriptors of Good Environmental Status.

The joint effectiveness of two or several measures is computed convolving effectiveness distributions of individual measures. As an example, Fig 2 shows the cumulative distributions of the effectiveness of two measures and how they jointly contribute to closing the gap with respect to one GES descriptor. Level 1 in gap closure represents the minimum threshold for reaching GES. Fig 3 demonstrates the gain in environmental quality with respect to the expected cost of a large number of alternative combinations of measures (about 570,000). This set of alternative PoMs includes a candidate PoM for which the measures have been selected based on Table 7, as well as candidates for which the measures have been selected randomly. As expected, the highest overall effectiveness is achieved by selecting all measures for inclusion in the PoM. On the other hand, total cost and environmental gain vary considerably across alternative combinations of measures, thus providing space for economic analysis. The efficient frontier, comprised of those combinations of measures that provide the best performance at each budget level, encompasses the candidate solutions to be offered for consideration to the decision makers. The overall performance of the optimal PoM increases with the budget available, but includes several smaller and one significant jump. The pattern seen in Fig 3, which resembles two overlapping leaves, is a result of one expensive measure (M17: Promote LNG as fuel for ships and provide the necessary infrastructure), which, if included in the PoM, increases the expected cost significantly but contributes only modestly to achieving GES.

**Fig 4:**
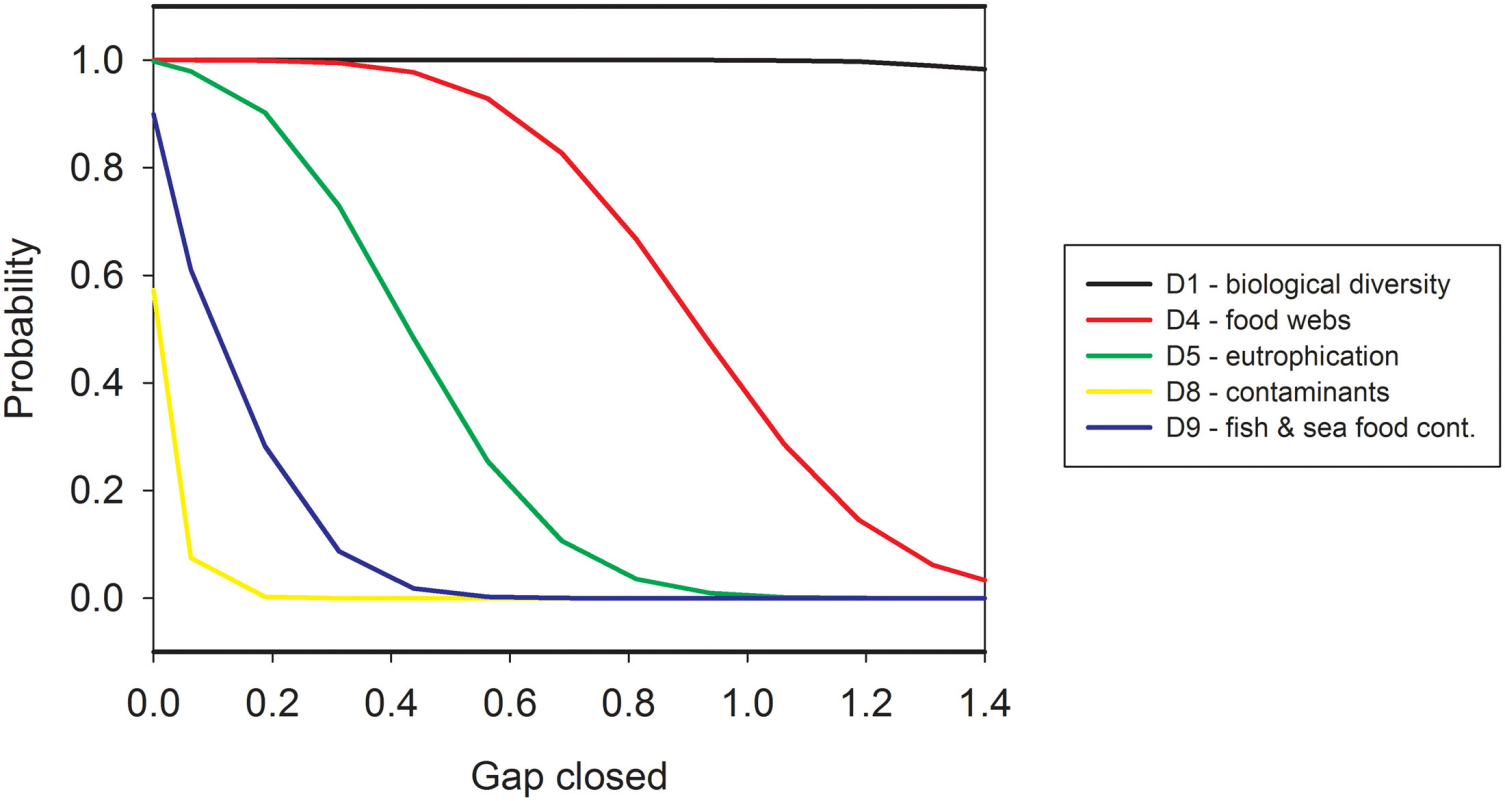
Cumulative probability for closing the gap for those five descriptors that currently fall short of GES assuming that all candidate measures were implemented.

Fig 4 shows the joint effects of measures on achieving GES where all 31 measures are implemented. In this case, it is quite likely that the gap between the desired and present state will be closed with respect to GES descriptor D1 (biodiversity). It is also possible that the objective of GES descriptor D4 (food webs) will be met. In the case of the other GES descriptors, it is less likely that the objectives will be met. Clearly, the number and effectiveness of the candidate measures (step 5 in Fig 1) has been too small. For eutrophication (D5), the difficulty of closing the gap is partly attributable to the time lags in the effects of nutrient abatement measures on water quality. In particular, the impacts of measures to reduce nutrient loads from agricultural lands may appear only after long delays [43, 44]. Furthermore, reducing excess nutrient loads is ultimately a trans-boundary problem, whose solution requires international cooperation [45]. It is also unlikely that the gap will be closed in the case of reducing contaminants (GES descriptors D8 and D9). The possibilities of boosting the rate at which existing toxic substances are removed from the marine ecosystem and food webs are rather limited, and the time horizon for improvements goes far beyond that of the first cycle of MSFD implementation.

### Cost-effective Programme of Measures

If there is no budget constraint, it is optimal to select all the measures for inclusion in the PoM (Table 7). However, with a budget constraint, it is reasonable, as demonstrated in Fig 3, to select the PoM more discriminatingly. Table 8 shows the cost-effective combinations of measures for various budget constraints. Success in closing the focal gap in environmental status is described in terms of a 90-percent confidence interval. Here we focus on those GES descriptors for which the state of the marine environment is seen as falling short of GES, i.e., *γ*_*j*_ = 1for j = 1,4,5,8,9 and *γ*_*j*_ = 0  for *j* = 2,3,6,7,10 and 11. In line with Ojaveer and Eero [46], equal weights have been assigned to those descriptors that show deviations from GES. The gaps, or the relative distance from GES, are assumed to be the same for those descriptors, i.e., *δ*_1_ = *δ*_4_ = *δ*_5_ = *δ*_8_ = *δ*_9_. A linear utility function is assumed. It is also assumed that effects of the measures are independent and that they do not have antagonistic or synergistic effects on each other.

**Table 8:** Cost-effective combinations of measures to narrow the existing gaps with different budget constraints.

		90% confidence interval for closing the gap				
Budget limit M €	Number of measures (measures included)	D1	D4	D5	D8	D9
20	21 measures (1,2,8,10, 12–15, 18–29, 31)	0.9–1.8	0.42–1.2	0.01–0.44	0–0.06	0–0.38
50	26 measures (1–3, 5, 8, 10–16, 18–29, 30–31)	1.2–2.2	0.46–1.2	0.06–0.66	0–0.11	0–0.38
90	29 measures (1–5, 7–16, 18–31)	1.5–2.6	0.50–1.3	0.08–0.71	0–0.11	0–0.38
unlimited^1^	31 measures (all measures)	1.5–2.6	0.52–1.3	0.11–0.78	0–0.11	0–0.38

^1^ The expected costs of implementing all 31 measures are 136.2 MEUR

The response surface in Fig 3 and the results in Table 8 imply that even with a limited budget it is possible to achieve a considerable proportion of the maximum joint effects. A budget constraint of 20 MEUR allows for implementation of 20 of the 31 new measures. The average cost of measures included in this candidate PoM is 1.0 MEUR, as compared to an average cost of 4.4 MEUR for all 31 measures. All 11 measures closing the gap for toxicants (D8, D9) are included in the low-budget PoMs. At a 90 MEUR budget constraint, almost full effectiveness is reached with respect to all GES descriptors. This PoM candidate would exclude two measures: M6 (A study of coastal species fisheries management and its efficiency), which affects only D3, and M17 (Promote LNG as fuel for ships and provide the necessary infrastructure), which is the most expensive measure (50 MEUR).

### Solutions for alternative problem formulations

All the computations can be repeated as soon as new candidate measures are identified or new data on the effectiveness or costs of measures become available. Moreover, the analysis is easily updated for new knowledge on the relative severity of the gaps (scaling factor *δ*), society’s preferences regarding different GES descriptors (scaling factor *γ*) or any other component of the modelling framework. The framework also allows for considering the problem of a risk-averse decision maker who, instead of looking at expected outcomes, may require that GES be pursued in the case of certain core descriptors for which the probability of success is higher. Such an objective may be attributed to the quest for a “safe minimum standard”. In addition, the model allows the societal utility function to be concave, rather than linear, and to include discontinuities.

We conducted sensitivity analyses for some of the parameter values and problem formulations. As a general finding, different attitudes towards the risk involved and form of the utility function had only a small impact on the optimal combination of measures. For these particular sets of alternative measures, the most promising measures—those characterized by low or moderate cost and moderate or high impact—came to form part of the optimal solutions irrespective of the problem formulation. Inclusion of a large number of low-cost measures in the PoM was found to be desirable both for lightening the left tail of the effectiveness distribution as well as for increasing the expected joint effect.

As an example of a discontinuous utility function, we studied the case where an analyst or decision maker wishes to close the gaps but disregards any further improvement. With a 50 MEUR budget constraint, the optimal solution was quite similar to the solution maximizing the joint effect (second row in Table 8). The number of measures included in the PoM was 25 (instead of 26). One measure was added (M9) and two removed (M8 and M11) to shift the emphasis towards better meeting a safe minimum standard in the case of GES descriptors D4 and D5. For a corresponding variant with a concave utility function,

$$\underset{\left\{x_{i},i=1,\ldots ,n\right\}}{\max}\sum_{j=1}^{m}\gamma_{j}*1.22\left\{1-e^{-1.84\text{E}\left(\sum_{i=1}^{n}\delta_{j}f_{ij}x_{i}\right)}\right\}.$$

the optimal solution with the 50 MEUR budget constraint was determined; this included 27 measures and deviated from the optimal solution using a linear utility function such that measures M4 and M7 were added to the PoM and measure M11 was removed from it.

## Discussion

The requirements made of CEA—that it accord with the ecosystem approach, accommodate the multi-dimensional environmental objective of the MSFD and have high policy relevance—call for pragmatic approaches and modeling techniques. This paper provides one approach for numerically analyzing and determining the cost-effective candidate PoMs for the national implementation of the MSFD. The numerical results of the framework allow the analyst to produce cost-effectiveness rankings of the measures, develop economically sound sets of measures under a variety of budget constraints and study the trade-offs associated with different policy objectives. Probabilistic definitions of costs and effects transparently demonstrate the underlying uncertainties and allow analyses from different risk perspectives, a feature lacking in CEA analyses of the WFD [15]. The framework is flexible with respect to data acquisition: the cause-effect-cost links between the measures and the focal environmental objective can be parameterized using models, statistics, expert knowledge, or a combination of these. The framework was designed to allow a CEA to be completed using limited data and resources in order to meet the urgent needs of the national marine strategy, but also to be flexible enough to be later amended with new sets of data or modelling components. It may be expected that the expert-based assessments capable of gathering large amounts of existing data on the effects and costs of management measures will pave the way for detailed future data collection and modelling [47]. One future improvement might be to replace the somewhat resource-intensive group interviews by an electronic survey questionnaire.

Our results demonstrate that for Finnish marine waters the target year of the MSFD– 2020 –comes too early for achieving GES with respect to all descriptors. The noticeably low effectiveness of some management measures with respect to the environmental target does not mean that the measures are insignificant, but rather that their implementation takes more time and that there is a time lag in the marine environment responding to them. Dynamic analyses allowing for the description of the effectiveness and costs of measures as trajectories rather than short-term estimates would allow for a more realistic ranking of alternative management measures.

In Finland, the PoM was prepared in expert working groups through collaboration between a socio-economic sub-group and five other thematic sub-groups, which focused on eutrophication, hazardous substances, marine resources, maritime transport and biodiversity, respectively. Due to administrative time constraints, the socio-economic group started its work much later than the others. As a consequence, the number of iterations in which information about the approach and results of CEA were exchanged between the socio-economic and other groups was limited. The central lesson learned from the process, expressed by several members of the various thematic groups, was that all groups should have started their work simultaneously. This would have enabled early communication and exchange of ideas about the methodological choices, administrative constraints, basic ideas of systems thinking and possible budget constraints pertaining to the PoM.

Developing a marine strategy, as required by the MSFD, is an iterative process cutting across different sectors of environmental administration and involving experts with varied academic backgrounds. Fruitful interdisciplinary collaboration requires that participants exhibit a certain level of openness and effort and that the organization provide opportunities and time for exchanging ideas. The basic idea of applying CEA to environmental policies is straightforward and can be easily adopted by specialists in various disciplines [1]. In fact, the major challenges of applying CEA to environmental improvements fall in the fields of the natural and technical sciences and are associated with the assessment of the current state of environment and the impacts of measures rather than with economic complexities. The contribution of economics can be seen as providing the systems thinking required to describe the socio-ecological system and the interaction between humans and other parts of the catchment-cost-marine system. In addition, economics plays an important role in determining the socially optimal level of marine protection efforts and the design of market-based policy instruments.

Given all the assumptions that have had to be made, our numerical results must be interpreted with care. Due to the limited time available for data acquisition and the large number of candidate measures, it was not possible to consider potential antagonistic and synergetic impacts of measures. Instead, it was assumed that all measures are mutually independent and additive. However, if measures contribute to achieving specific, narrow elements of a GES descriptor, their impacts may not be directly additive. This is often the case with the descriptors pertaining to biodiversity. Ideally, biodiversity is described in terms of a multidimensional matrix covering relevant elements of biodiversity, such as the groups of flora and fauna specified at appropriate spatial and temporal scales. The corresponding GES descriptor should, in some balanced manner, synthesize these elements into a single value [see [19] for a review of different approaches to aggregate indicators]. This caveat was, to some degree, acknowledged in the process of eliciting effectiveness: the measures that considered several elements of biodiversity and protected both species and their habitats were assessed as being more effective than measures protecting species only. The assessments were scaled to earlier assessments addressing the issue of the number of species needing protection and the ranking of the species groups based on their vulnerability [17]. However, it is still likely that our results overestimate the joint effect of several measures on the GES descriptors, in particular species diversity. In this light, the probability distribution for GES descriptor D1 (biodiversity) and most likely that for GES descriptor D4 (food webs) as well are overestimates and serve as “upper bounds” of the effects.

This paper provides a framework for the CEA needed in producing Finland's PoM as required by the MSFD. However, the Directive also calls for cost-benefit analyses to identify potentially disproportionate costs. The framework presented here can be amended to incorporate cost-benefit analysis if estimates of the economic benefits of water protection measures are available in monetary terms. Ideally, such information could be obtained through an extensive valuation study. However, a pragmatic solution for the estimation of a utility function describing the economic benefits of the protection measures would be to apply the existing knowledge on citizens’ preferences regarding marine protection through benefit transfers [45, 48–53]. For those descriptors for which no studies exist, expert elicitation could be applied.

## Conclusions

In this study we introduce a framework for probabilistic and holistic cost-effectiveness analysis that makes it possible to consider the economic efficiency of management measures and to develop the least-cost set of measures to reach the multidimensional environmental objective. The framework is illustrated by applying it in choosing the Programme of Measures in Finland to reach the targets of Marine Strategy Framework Directive. To our knowledge, this is the first attempt to numerically develop the cost-effective Programmes of Measures, required as part of the national implementation of the MSFD. The present analysis serves as an example of how economic analysis can be applied in assessing water protection programs in cases where the environmental objective and thus the means to achieve that target are multidimensional. The main challenge in conducting cost-effectiveness analysis for marine protection turned out to be the acquisition of data on the marginal costs and multiple effects of measures. Engaging systems thinking early on in an iterative process for developing candidate PoMs and carrying out the other phases of implementing the MSFD could yield carefully elaborated marine strategies in which the scarce resources available are efficiently allocated.

## Supporting Information

S1 Table Probability of overall cost for the period: 2016–2022. (DOCX)

S2 Table Probability distributions for gap closure. (DOCX)
