# Supplementary material for: Cost-Effective Marine Protection - A Pragmatic Approach
Source: PLoS One. 2016 Jan 11;11(1):e0147085. doi: 10.1371/journal.pone.0147085 (PMC4709167; doi:10.1371/journal.pone.0147085)
Supplement: S1 Table — (DOCX) [file pone.0147085.s001.docx]

|  |  |  |  |  |  |  |  |
| --- | --- | --- | --- | --- | --- | --- | --- |
| Measure | 0-0.1 M€ | 0.1-0.5 M€ | 0.5-1 M€ | 1-5 M€ | 5-10 M€ | 10-50 M€ | >50 M€ |
| M1 | 0 | 0.25 | 0.25 | 0.25 | 0.25 | 0 | 0 |
| M2 | 0.1 | 0.4 | 0.4 | 0.1 | 0 | 0 | 0 |
| M3 | 0 | 0.25 | 0.25 | 0.25 | 0.25 | 0 | 0 |
| M4 | 0 | 0 | 0.1 | 0.4 | 0.4 | 0.1 | 0 |
| M5 | 0 | 0.5 | 0.5 | 0 | 0 | 0 | 0 |
| M6 | 0.8 | 0.1 | 0.1 | 0 | 0 | 0 | 0 |
| M7 | 0 | 0 | 0 | 0.1 | 0.7 | 0.2 | 0 |
| M8 | 0 | 0.1 | 0.4 | 0.4 | 0.1 | 0 | 0 |
| M9 | 0 | 0 | 0 | 0.2 | 0.3 | 0.3 | 0.2 |
| M10 | 0 | 0 | 0.5 | 0.3 | 0.2 | 0 | 0 |
| M11 | 0 | 0 | 0 | 0 | 0.5 | 0.5 | 0 |
| M12 | 0.6 | 0.4 | 0 | 0 | 0 | 0 | 0 |
| M13 | 0 | 0.2 | 0.7 | 0.1 | 0 | 0 | 0 |
| M14 | 0.1 | 0.4 | 0.4 | 0.1 | 0 | 0 | 0 |
| M15 | 0 | 0.2 | 0.4 | 0.4 | 0 | 0 | 0 |
| M16 | 0 | 0 | 0.4 | 0.5 | 0.1 | 0 | 0 |
| M17 | 0 | 0 | 0 | 0 | 0 | 0 | 1 |
| M18 | 0 | 0.3 | 0.4 | 0.3 | 0 | 0 | 0 |
| M19 | 0.3 | 0.6 | 0.1 | 0 | 0 | 0 | 0 |
| M20 | 0.1 | 0.5 | 0.4 | 0 | 0 | 0 | 0 |
| M21 | 0 | 0.9 | 0.1 | 0 | 0 | 0 | 0 |
| M22 | 0 | 0.4 | 0.5 | 0.1 | 0 | 0 | 0 |
| M23 | 0.7 | 0.3 | 0 | 0 | 0 | 0 | 0 |
| M24 | 0.7 | 0.3 | 0 | 0 | 0 | 0 | 0 |
| M25 | 0 | 0.2 | 0.6 | 0.2 | 0 | 0 | 0 |
| M26 | 0 | 0.7 | 0.3 | 0 | 0 | 0 | 0 |
| M27 | 0 | 0.7 | 0.3 | 0 | 0 | 0 | 0 |
| M28 | 0 | 0.2 | 0.4 | 0.3 | 0.1 | 0 | 0 |
| M29 | 0 | 0.3 | 0.6 | 0.1 | 0 | 0 | 0 |
| M30 | 0.1 | 0.7 | 0.2 | 0 | 0 | 0 | 0 |
| M31 | 1 | 0 | 0 | 0 | 0 | 0 | 0 |
