# Supplementary material for: Cost-Effective Marine Protection - A Pragmatic Approach
Source: PLoS One. 2016 Jan 11;11(1):e0147085. doi: 10.1371/journal.pone.0147085 (PMC4709167; doi:10.1371/journal.pone.0147085)
Supplement: S2 Table — (DOCX) [file pone.0147085.s002.docx]

| Measure | Gap clos., prob. | D1 | D2 | D3 | D4 | D5 | D6 | D7 | D8 | D9 | D10 | D11 |
| --- | --- | --- | --- | --- | --- | --- | --- | --- | --- | --- | --- | --- |
| M1 | 0% | 0.8 | 1 | 1 | 0.8 | 0.2 | 0.9 | 1 | 1 | 1 | 1 | 1 |
|  | 0-12.5% | 0.2 | 0 | 0 | 0.2 | 0.7 | 0.1 | 0 | 0 | 0 | 0 | 0 |
|  | 12.5-25% | 0 | 0 | 0 | 0 | 0.1 | 0 | 0 | 0 | 0 | 0 | 0 |
|  | 25-50% | 0 | 0 | 0 | 0 | 0 | 0 | 0 | 0 | 0 | 0 | 0 |
|  | 50-75% | 0 | 0 | 0 | 0 | 0 | 0 | 0 | 0 | 0 | 0 | 0 |
|  | 75-100% | 0 | 0 | 0 | 0 | 0 | 0 | 0 | 0 | 0 | 0 | 0 |
|  | >100% | 0 | 0 | 0 | 0 | 0 | 0 | 0 | 0 | 0 | 0 | 0 |
| M2 | 0% | 0.4 | 1 | 1 | 0.4 | 0.2 | 0.8 | 1 | 1 | 1 | 1 | 1 |
|  | 0-12.5% | 0.6 | 0 | 0 | 0.6 | 0.4 | 0.2 | 0 | 0 | 0 | 0 | 0 |
|  | 12.5-25% | 0 | 0 | 0 | 0 | 0.3 | 0 | 0 | 0 | 0 | 0 | 0 |
|  | 25-50% | 0 | 0 | 0 | 0 | 0.1 | 0 | 0 | 0 | 0 | 0 | 0 |
|  | 50-75% | 0 | 0 | 0 | 0 | 0 | 0 | 0 | 0 | 0 | 0 | 0 |
|  | 75-100% | 0 | 0 | 0 | 0 | 0 | 0 | 0 | 0 | 0 | 0 | 0 |
|  | >100% | 0 | 0 | 0 | 0 | 0 | 0 | 0 | 0 | 0 | 0 | 0 |
| M3 | 0% | 0.93 | 1 | 1 | 0.93 | 0.4 | 1 | 1 | 1 | 1 | 1 | 1 |
|  | 0-12.5% | 0.07 | 0 | 0 | 0.07 | 0.6 | 0 | 0 | 0 | 0 | 0 | 0 |
|  | 12.5-25% | 0 | 0 | 0 | 0 | 0 | 0 | 0 | 0 | 0 | 0 | 0 |
|  | 25-50% | 0 | 0 | 0 | 0 | 0 | 0 | 0 | 0 | 0 | 0 | 0 |
|  | 50-75% | 0 | 0 | 0 | 0 | 0 | 0 | 0 | 0 | 0 | 0 | 0 |
|  | 75-100% | 0 | 0 | 0 | 0 | 0 | 0 | 0 | 0 | 0 | 0 | 0 |
|  | >100% | 0 | 0 | 0 | 0 | 0 | 0 | 0 | 0 | 0 | 0 | 0 |
| M4 | 0% | 0.6 | 1 | 1 | 0.8 | 0.8 | 1 | 1 | 1 | 1 | 1 | 1 |
|  | 0-12.5% | 0.4 | 0 | 0 | 0.2 | 0.2 | 0 | 0 | 0 | 0 | 0 | 0 |
|  | 12.5-25% | 0 | 0 | 0 | 0 | 0 | 0 | 0 | 0 | 0 | 0 | 0 |
|  | 25-50% | 0 | 0 | 0 | 0 | 0 | 0 | 0 | 0 | 0 | 0 | 0 |
|  | 50-75% | 0 | 0 | 0 | 0 | 0 | 0 | 0 | 0 | 0 | 0 | 0 |
|  | 75-100% | 0 | 0 | 0 | 0 | 0 | 0 | 0 | 0 | 0 | 0 | 0 |
|  | >100% | 0 | 0 | 0 | 0 | 0 | 0 | 0 | 0 | 0 | 0 | 0 |
| M5 | 0% | 0.97 | 1 | 1 | 0.98 | 0.7 | 0.99 | 1 | 1 | 1 | 1 | 1 |
|  | 0-12.5% | 0.03 | 0 | 0 | 0.02 | 0.3 | 0.01 | 0 | 0 | 0 | 0 | 0 |
|  | 12.5-25% | 0 | 0 | 0 | 0 | 0 | 0 | 0 | 0 | 0 | 0 | 0 |
|  | 25-50% | 0 | 0 | 0 | 0 | 0 | 0 | 0 | 0 | 0 | 0 | 0 |
|  | 50-75% | 0 | 0 | 0 | 0 | 0 | 0 | 0 | 0 | 0 | 0 | 0 |
|  | 75-100% | 0 | 0 | 0 | 0 | 0 | 0 | 0 | 0 | 0 | 0 | 0 |
|  | >100% | 0 | 0 | 0 | 0 | 0 | 0 | 0 | 0 | 0 | 0 | 0 |
| M6 | 0% | 1 | 1 | 0.9 | 1 | 1 | 1 | 1 | 1 | 1 | 1 | 1 |
|  | 0-12.5% | 0 | 0 | 0.1 | 0 | 0 | 0 | 0 | 0 | 0 | 0 | 0 |
|  | 12.5-25% | 0 | 0 | 0 | 0 | 0 | 0 | 0 | 0 | 0 | 0 | 0 |
|  | 25-50% | 0 | 0 | 0 | 0 | 0 | 0 | 0 | 0 | 0 | 0 | 0 |
|  | 50-75% | 0 | 0 | 0 | 0 | 0 | 0 | 0 | 0 | 0 | 0 | 0 |
|  | 75-100% | 0 | 0 | 0 | 0 | 0 | 0 | 0 | 0 | 0 | 0 | 0 |
|  | >100% | 0 | 0 | 0 | 0 | 0 | 0 | 0 | 0 | 0 | 0 | 0 |
| M7 | 0% | 0 | 1 | 0 | 1 | 1 | 1 | 1 | 1 | 1 | 1 | 1 |
|  | 0-12.5% | 0.9 | 0 | 0.5 | 0 | 0 | 0 | 0 | 0 | 0 | 0 | 0 |
|  | 12.5-25% | 0.1 | 0 | 0.4 | 0 | 0 | 0 | 0 | 0 | 0 | 0 | 0 |
|  | 25-50% | 0 | 0 | 0.1 | 0 | 0 | 0 | 0 | 0 | 0 | 0 | 0 |
|  | 50-75% | 0 | 0 | 0 | 0 | 0 | 0 | 0 | 0 | 0 | 0 | 0 |
|  | 75-100% | 0 | 0 | 0 | 0 | 0 | 0 | 0 | 0 | 0 | 0 | 0 |
|  | >100% | 0 | 0 | 0 | 0 | 0 | 0 | 0 | 0 | 0 | 0 | 0 |
| M8 | 0% | 0.3 | 1 | 1 | 1 | 1 | 1 | 1 | 1 | 1 | 1 | 1 |
|  | 0-12.5% | 0.7 | 0 | 0 | 0 | 0 | 0 | 0 | 0 | 0 | 0 | 0 |
|  | 12.5-25% | 0 | 0 | 0 | 0 | 0 | 0 | 0 | 0 | 0 | 0 | 0 |
|  | 25-50% | 0 | 0 | 0 | 0 | 0 | 0 | 0 | 0 | 0 | 0 | 0 |
|  | 50-75% | 0 | 0 | 0 | 0 | 0 | 0 | 0 | 0 | 0 | 0 | 0 |
|  | 75-100% | 0 | 0 | 0 | 0 | 0 | 0 | 0 | 0 | 0 | 0 | 0 |
|  | >100% | 0 | 0 | 0 | 0 | 0 | 0 | 0 | 0 | 0 | 0 | 0 |
| M9 | 0% | 0.2 | 0.9 | 0.3 | 0.6 | 0.95 | 0.1 | 0.9 | 1 | 1 | 1 | 0.2 |
|  | 0-12.5% | 0.4 | 0.1 | 0.5 | 0.4 | 0.05 | 0.5 | 0.1 | 0 | 0 | 0 | 0.7 |
|  | 12.5-25% | 0.3 | 0 | 0.2 | 0 | 0 | 0.3 | 0 | 0 | 0 | 0 | 0.1 |
|  | 25-50% | 0.1 | 0 | 0 | 0 | 0 | 0.1 | 0 | 0 | 0 | 0 | 0 |
|  | 50-75% | 0 | 0 | 0 | 0 | 0 | 0 | 0 | 0 | 0 | 0 | 0 |
|  | 75-100% | 0 | 0 | 0 | 0 | 0 | 0 | 0 | 0 | 0 | 0 | 0 |
|  | >100% | 0 | 0 | 0 | 0 | 0 | 0 | 0 | 0 | 0 | 0 | 0 |
| M10 | 0% | 0 | 1 | 0 | 1 | 1 | 0.1 | 1 | 1 | 1 | 0.5 | 0.2 |
|  | 0-12.5% | 0.2 | 0 | 0.5 | 0 | 0 | 0.6 | 0 | 0 | 0 | 0.5 | 0.8 |
|  | 12.5-25% | 0.6 | 0 | 0.5 | 0 | 0 | 0.3 | 0 | 0 | 0 | 0 | 0 |
|  | 25-50% | 0.2 | 0 | 0 | 0 | 0 | 0 | 0 | 0 | 0 | 0 | 0 |
|  | 50-75% | 0 | 0 | 0 | 0 | 0 | 0 | 0 | 0 | 0 | 0 | 0 |
|  | 75-100% | 0 | 0 | 0 | 0 | 0 | 0 | 0 | 0 | 0 | 0 | 0 |
|  | >100% | 0 | 0 | 0 | 0 | 0 | 0 | 0 | 0 | 0 | 0 | 0 |
| M11 | 0% | 0 | 0.6 | 0.5 | 0.7 | 1 | 0.3 | 1 | 1 | 1 | 1 | 0.8 |
|  | 0-12.5% | 0.1 | 0.4 | 0.5 | 0.3 | 0 | 0.7 | 0 | 0 | 0 | 0 | 0.2 |
|  | 12.5-25% | 0.5 | 0 | 0 | 0 | 0 | 0 | 0 | 0 | 0 | 0 | 0 |
|  | 25-50% | 0.4 | 0 | 0 | 0 | 0 | 0 | 0 | 0 | 0 | 0 | 0 |
|  | 50-75% | 0 | 0 | 0 | 0 | 0 | 0 | 0 | 0 | 0 | 0 | 0 |
|  | 75-100% | 0 | 0 | 0 | 0 | 0 | 0 | 0 | 0 | 0 | 0 | 0 |
|  | >100% | 0 | 0 | 0 | 0 | 0 | 0 | 0 | 0 | 0 | 0 | 0 |
| M12 | 0% | 0.3 | 0.8 | 1 | 1 | 1 | 0.2 | 1 | 1 | 1 | 0.4 | 0.7 |
|  | 0-12.5% | 0.6 | 0.2 | 0 | 0 | 0 | 0.8 | 0 | 0 | 0 | 0.6 | 0.3 |
|  | 12.5-25% | 0.1 | 0 | 0 | 0 | 0 | 0 | 0 | 0 | 0 | 0 | 0 |
|  | 25-50% | 0 | 0 | 0 | 0 | 0 | 0 | 0 | 0 | 0 | 0 | 0 |
|  | 50-75% | 0 | 0 | 0 | 0 | 0 | 0 | 0 | 0 | 0 | 0 | 0 |
|  | 75-100% | 0 | 0 | 0 | 0 | 0 | 0 | 0 | 0 | 0 | 0 | 0 |
|  | >100% | 0 | 0 | 0 | 0 | 0 | 0 | 0 | 0 | 0 | 0 | 0 |
| M13 | 0% | 0.3 | 1 | 1 | 0.2 | 1 | 1 | 1 | 1 | 1 | 1 | 1 |
|  | 0-12.5% | 0.6 | 0 | 0 | 0.6 | 0 | 0 | 0 | 0 | 0 | 0 | 0 |
|  | 12.5-25% | 0.1 | 0 | 0 | 0.2 | 0 | 0 | 0 | 0 | 0 | 0 | 0 |
|  | 25-50% | 0 | 0 | 0 | 0 | 0 | 0 | 0 | 0 | 0 | 0 | 0 |
|  | 50-75% | 0 | 0 | 0 | 0 | 0 | 0 | 0 | 0 | 0 | 0 | 0 |
|  | 75-100% | 0 | 0 | 0 | 0 | 0 | 0 | 0 | 0 | 0 | 0 | 0 |
|  | >100% | 0 | 0 | 0 | 0 | 0 | 0 | 0 | 0 | 0 | 0 | 0 |
| M14 | 0% | 0.5 | 1 | 1 | 0.9 | 0.9 | 0.1 | 0.9 | 1 | 1 | 1 | 1 |
|  | 0-12.5% | 0.4 | 0 | 0 | 0.1 | 0.1 | 0.7 | 0.1 | 0 | 0 | 0 | 0 |
|  | 12.5-25% | 0.1 | 0 | 0 | 0 | 0 | 0.2 | 0 | 0 | 0 | 0 | 0 |
|  | 25-50% | 0 | 0 | 0 | 0 | 0 | 0 | 0 | 0 | 0 | 0 | 0 |
|  | 50-75% | 0 | 0 | 0 | 0 | 0 | 0 | 0 | 0 | 0 | 0 | 0 |
|  | 75-100% | 0 | 0 | 0 | 0 | 0 | 0 | 0 | 0 | 0 | 0 | 0 |
|  | >100% | 0 | 0 | 0 | 0 | 0 | 0 | 0 | 0 | 0 | 0 | 0 |
| M15 | 0% | 0.67 | 1 | 0.9 | 0.8 | 1 | 1 | 1 | 0.5 | 0.9 | 1 | 1 |
|  | 0-12.5% | 0.33 | 0 | 0.1 | 0.2 | 0 | 0 | 0 | 0.5 | 0.1 | 0 | 0 |
|  | 12.5-25% | 0 | 0 | 0 | 0 | 0 | 0 | 0 | 0 | 0 | 0 | 0 |
|  | 25-50% | 0 | 0 | 0 | 0 | 0 | 0 | 0 | 0 | 0 | 0 | 0 |
|  | 50-75% | 0 | 0 | 0 | 0 | 0 | 0 | 0 | 0 | 0 | 0 | 0 |
|  | 75-100% | 0 | 0 | 0 | 0 | 0 | 0 | 0 | 0 | 0 | 0 | 0 |
|  | >100% | 0 | 0 | 0 | 0 | 0 | 0 | 0 | 0 | 0 | 0 | 0 |
| M16 | 0% | 0.95 | 1 | 0.95 | 0.95 | 0.5 | 1 | 1 | 1 | 1 | 1 | 1 |
|  | 0-12.5% | 0.05 | 0 | 0.05 | 0.05 | 0.5 | 0 | 0 | 0 | 0 | 0 | 0 |
|  | 12.5-25% | 0 | 0 | 0 | 0 | 0 | 0 | 0 | 0 | 0 | 0 | 0 |
|  | 25-50% | 0 | 0 | 0 | 0 | 0 | 0 | 0 | 0 | 0 | 0 | 0 |
|  | 50-75% | 0 | 0 | 0 | 0 | 0 | 0 | 0 | 0 | 0 | 0 | 0 |
|  | 75-100% | 0 | 0 | 0 | 0 | 0 | 0 | 0 | 0 | 0 | 0 | 0 |
|  | >100% | 0 | 0 | 0 | 0 | 0 | 0 | 0 | 0 | 0 | 0 | 0 |
| M17 | 0% | 0.95 | 1 | 0.95 | 0.95 | 0.5 | 1 | 1 | 1 | 1 | 1 | 1 |
|  | 0-12.5% | 0.05 | 0 | 0.05 | 0.05 | 0.5 | 0 | 0 | 0 | 0 | 0 | 0 |
|  | 12.5-25% | 0 | 0 | 0 | 0 | 0 | 0 | 0 | 0 | 0 | 0 | 0 |
|  | 25-50% | 0 | 0 | 0 | 0 | 0 | 0 | 0 | 0 | 0 | 0 | 0 |
|  | 50-75% | 0 | 0 | 0 | 0 | 0 | 0 | 0 | 0 | 0 | 0 | 0 |
|  | 75-100% | 0 | 0 | 0 | 0 | 0 | 0 | 0 | 0 | 0 | 0 | 0 |
|  | >100% | 0 | 0 | 0 | 0 | 0 | 0 | 0 | 0 | 0 | 0 | 0 |
| M18 | 0% | 0.5 | 1 | 0.5 | 0.5 | 1 | 1 | 1 | 1 | 1 | 1 | 0.2 |
|  | 0-12.5% | 0.5 | 0 | 0.5 | 0.5 | 0 | 0 | 0 | 0 | 0 | 0 | 0.6 |
|  | 12.5-25% | 0 | 0 | 0 | 0 | 0 | 0 | 0 | 0 | 0 | 0 | 0.2 |
|  | 25-50% | 0 | 0 | 0 | 0 | 0 | 0 | 0 | 0 | 0 | 0 | 0 |
|  | 50-75% | 0 | 0 | 0 | 0 | 0 | 0 | 0 | 0 | 0 | 0 | 0 |
|  | 75-100% | 0 | 0 | 0 | 0 | 0 | 0 | 0 | 0 | 0 | 0 | 0 |
|  | >100% | 0 | 0 | 0 | 0 | 0 | 0 | 0 | 0 | 0 | 0 | 0 |
| M19 | 0% | 0.3 | 1 | 0.9 | 0.2 | 1 | 1 | 1 | 1 | 1 | 1 | 0 |
|  | 0-12.5% | 0.7 | 0 | 0.1 | 0.8 | 0 | 0 | 0 | 0 | 0 | 0 | 0 |
|  | 12.5-25% | 0 | 0 | 0 | 0 | 0 | 0 | 0 | 0 | 0 | 0 | 0.2 |
|  | 25-50% | 0 | 0 | 0 | 0 | 0 | 0 | 0 | 0 | 0 | 0 | 0.6 |
|  | 50-75% | 0 | 0 | 0 | 0 | 0 | 0 | 0 | 0 | 0 | 0 | 0.2 |
|  | 75-100% | 0 | 0 | 0 | 0 | 0 | 0 | 0 | 0 | 0 | 0 | 0 |
|  | >100% | 0 | 0 | 0 | 0 | 0 | 0 | 0 | 0 | 0 | 0 | 0 |
| M20 | 0% | 0.2 | 1 | 1 | 1 | 1 | 1 | 1 | 1 | 1 | 1 | 0.2 |
|  | 0-12.5% | 0.8 | 0 | 0 | 0 | 0 | 0 | 0 | 0 | 0 | 0 | 0.8 |
|  | 12.5-25% | 0 | 0 | 0 | 0 | 0 | 0 | 0 | 0 | 0 | 0 | 0 |
|  | 25-50% | 0 | 0 | 0 | 0 | 0 | 0 | 0 | 0 | 0 | 0 | 0 |
|  | 50-75% | 0 | 0 | 0 | 0 | 0 | 0 | 0 | 0 | 0 | 0 | 0 |
|  | 75-100% | 0 | 0 | 0 | 0 | 0 | 0 | 0 | 0 | 0 | 0 | 0 |
|  | >100% | 0 | 0 | 0 | 0 | 0 | 0 | 0 | 0 | 0 | 0 | 0 |
| M21 | 0% | 0.5 | 1 | 0.5 | 0.4 | 1 | 1 | 1 | 1 | 0.7 | 0.1 | 1 |
|  | 0-12.5% | 0.5 | 0 | 0.5 | 0.6 | 0 | 0 | 0 | 0 | 0.3 | 0.8 | 0 |
|  | 12.5-25% | 0 | 0 | 0 | 0 | 0 | 0 | 0 | 0 | 0 | 0.1 | 0 |
|  | 25-50% | 0 | 0 | 0 | 0 | 0 | 0 | 0 | 0 | 0 | 0 | 0 |
|  | 50-75% | 0 | 0 | 0 | 0 | 0 | 0 | 0 | 0 | 0 | 0 | 0 |
|  | 75-100% | 0 | 0 | 0 | 0 | 0 | 0 | 0 | 0 | 0 | 0 | 0 |
|  | >100% | 0 | 0 | 0 | 0 | 0 | 0 | 0 | 0 | 0 | 0 | 0 |
| M22 | 0% | 0.7 | 1 | 0.7 | 0.6 | 1 | 1 | 1 | 1 | 0.9 | 0.2 | 1 |
|  | 0-12.5% | 0.3 | 0 | 0.3 | 0.4 | 0 | 0 | 0 | 0 | 0.1 | 0.7 | 0 |
|  | 12.5-25% | 0 | 0 | 0 | 0 | 0 | 0 | 0 | 0 | 0 | 0.1 | 0 |
|  | 25-50% | 0 | 0 | 0 | 0 | 0 | 0 | 0 | 0 | 0 | 0 | 0 |
|  | 50-75% | 0 | 0 | 0 | 0 | 0 | 0 | 0 | 0 | 0 | 0 | 0 |
|  | 75-100% | 0 | 0 | 0 | 0 | 0 | 0 | 0 | 0 | 0 | 0 | 0 |
|  | >100% | 0 | 0 | 0 | 0 | 0 | 0 | 0 | 0 | 0 | 0 | 0 |
| M23 | 0% | 0.8 | 1 | 0.8 | 0.7 | 1 | 1 | 1 | 1 | 0.95 | 0.2 | 1 |
|  | 0-12.5% | 0.2 | 0 | 0.2 | 0.3 | 0 | 0 | 0 | 0 | 0.05 | 0.8 | 0 |
|  | 12.5-25% | 0 | 0 | 0 | 0 | 0 | 0 | 0 | 0 | 0 | 0 | 0 |
|  | 25-50% | 0 | 0 | 0 | 0 | 0 | 0 | 0 | 0 | 0 | 0 | 0 |
|  | 50-75% | 0 | 0 | 0 | 0 | 0 | 0 | 0 | 0 | 0 | 0 | 0 |
|  | 75-100% | 0 | 0 | 0 | 0 | 0 | 0 | 0 | 0 | 0 | 0 | 0 |
|  | >100% | 0 | 0 | 0 | 0 | 0 | 0 | 0 | 0 | 0 | 0 | 0 |
| M24 | 0% | 0.9 | 1 | 0.9 | 0.8 | 1 | 1 | 1 | 1 | 0.9 | 0.5 | 1 |
|  | 0-12.5% | 0.1 | 0 | 0.1 | 0.2 | 0 | 0 | 0 | 0 | 0.1 | 0.5 | 0 |
|  | 12.5-25% | 0 | 0 | 0 | 0 | 0 | 0 | 0 | 0 | 0 | 0 | 0 |
|  | 25-50% | 0 | 0 | 0 | 0 | 0 | 0 | 0 | 0 | 0 | 0 | 0 |
|  | 50-75% | 0 | 0 | 0 | 0 | 0 | 0 | 0 | 0 | 0 | 0 | 0 |
|  | 75-100% | 0 | 0 | 0 | 0 | 0 | 0 | 0 | 0 | 0 | 0 | 0 |
|  | >100% | 0 | 0 | 0 | 0 | 0 | 0 | 0 | 0 | 0 | 0 | 0 |
| M25 | 0% | 0.4 | 1 | 0.4 | 0.3 | 1 | 1 | 1 | 1 | 0.6 | 0.1 | 1 |
|  | 0-12.5% | 0.6 | 0 | 0.6 | 0.7 | 0 | 0 | 0 | 0 | 0.4 | 0.6 | 0 |
|  | 12.5-25% | 0 | 0 | 0 | 0 | 0 | 0 | 0 | 0 | 0 | 0.3 | 0 |
|  | 25-50% | 0 | 0 | 0 | 0 | 0 | 0 | 0 | 0 | 0 | 0 | 0 |
|  | 50-75% | 0 | 0 | 0 | 0 | 0 | 0 | 0 | 0 | 0 | 0 | 0 |
|  | 75-100% | 0 | 0 | 0 | 0 | 0 | 0 | 0 | 0 | 0 | 0 | 0 |
|  | >100% | 0 | 0 | 0 | 0 | 0 | 0 | 0 | 0 | 0 | 0 | 0 |
| M26 | 0% | 0.9 | 1 | 0.9 | 0.8 | 1 | 1 | 1 | 1 | 0.9 | 0.5 | 1 |
|  | 0-12.5% | 0.1 | 0 | 0.1 | 0.2 | 0 | 0 | 0 | 0 | 0.1 | 0.5 | 0 |
|  | 12.5-25% | 0 | 0 | 0 | 0 | 0 | 0 | 0 | 0 | 0 | 0 | 0 |
|  | 25-50% | 0 | 0 | 0 | 0 | 0 | 0 | 0 | 0 | 0 | 0 | 0 |
|  | 50-75% | 0 | 0 | 0 | 0 | 0 | 0 | 0 | 0 | 0 | 0 | 0 |
|  | 75-100% | 0 | 0 | 0 | 0 | 0 | 0 | 0 | 0 | 0 | 0 | 0 |
|  | >100% | 0 | 0 | 0 | 0 | 0 | 0 | 0 | 0 | 0 | 0 | 0 |
| M27 | 0% | 0.9 | 1 | 0.7 | 0.8 | 1 | 1 | 1 | 1 | 0.9 | 0.5 | 1 |
|  | 0-12.5% | 0.1 | 0 | 0.3 | 0.2 | 0 | 0 | 0 | 0 | 0.1 | 0.5 | 0 |
|  | 12.5-25% | 0 | 0 | 0 | 0 | 0 | 0 | 0 | 0 | 0 | 0 | 0 |
|  | 25-50% | 0 | 0 | 0 | 0 | 0 | 0 | 0 | 0 | 0 | 0 | 0 |
|  | 50-75% | 0 | 0 | 0 | 0 | 0 | 0 | 0 | 0 | 0 | 0 | 0 |
|  | 75-100% | 0 | 0 | 0 | 0 | 0 | 0 | 0 | 0 | 0 | 0 | 0 |
|  | >100% | 0 | 0 | 0 | 0 | 0 | 0 | 0 | 0 | 0 | 0 | 0 |
| M28 | 0% | 0.3 | 1 | 0.3 | 0.2 | 1 | 1 | 1 | 1 | 0.5 | 0 | 1 |
|  | 0-12.5% | 0.7 | 0 | 0.7 | 0.8 | 0 | 0 | 0 | 0 | 0.5 | 0.7 | 0 |
|  | 12.5-25% | 0 | 0 | 0 | 0 | 0 | 0 | 0 | 0 | 0 | 0.3 | 0 |
|  | 25-50% | 0 | 0 | 0 | 0 | 0 | 0 | 0 | 0 | 0 | 0 | 0 |
|  | 50-75% | 0 | 0 | 0 | 0 | 0 | 0 | 0 | 0 | 0 | 0 | 0 |
|  | 75-100% | 0 | 0 | 0 | 0 | 0 | 0 | 0 | 0 | 0 | 0 | 0 |
|  | >100% | 0 | 0 | 0 | 0 | 0 | 0 | 0 | 0 | 0 | 0 | 0 |
| M29 | 0% | 0 | 1 | 1 | 0.5 | 1 | 1 | 0 | 1 | 1 | 1 | 1 |
|  | 0-12.5% | 1 | 0 | 0 | 0.5 | 0 | 0 | 0 | 0 | 0 | 0 | 0 |
|  | 12.5-25% | 0 | 0 | 0 | 0 | 0 | 0 | 0 | 0 | 0 | 0 | 0 |
|  | 25-50% | 0 | 0 | 0 | 0 | 0 | 0 | 0.5 | 0 | 0 | 0 | 0 |
|  | 50-75% | 0 | 0 | 0 | 0 | 0 | 0 | 0.5 | 0 | 0 | 0 | 0 |
|  | 75-100% | 0 | 0 | 0 | 0 | 0 | 0 | 0 | 0 | 0 | 0 | 0 |
|  | >100% | 0 | 0 | 0 | 0 | 0 | 0 | 0 | 0 | 0 | 0 | 0 |
| M30 | 0% | 1 | 1 | 1 | 1 | 1 | 1 | 1 | 0.9 | 0.9 | 1 | 1 |
|  | 0-12.5% | 0 | 0 | 0 | 0 | 0 | 0 | 0 | 0.1 | 0.1 | 0 | 0 |
|  | 12.5-25% | 0 | 0 | 0 | 0 | 0 | 0 | 0 | 0 | 0 | 0 | 0 |
|  | 25-50% | 0 | 0 | 0 | 0 | 0 | 0 | 0 | 0 | 0 | 0 | 0 |
|  | 50-75% | 0 | 0 | 0 | 0 | 0 | 0 | 0 | 0 | 0 | 0 | 0 |
|  | 75-100% | 0 | 0 | 0 | 0 | 0 | 0 | 0 | 0 | 0 | 0 | 0 |
|  | >100% | 0 | 0 | 0 | 0 | 0 | 0 | 0 | 0 | 0 | 0 | 0 |
| M31 | 0% | 1 | 1 | 1 | 1 | 1 | 1 | 1 | 0.95 | 0.95 | 1 | 1 |
|  | 0-12.5% | 0 | 0 | 0 | 0 | 0 | 0 | 0 | 0.05 | 0.05 | 0 | 0 |
|  | 12.5-25% | 0 | 0 | 0 | 0 | 0 | 0 | 0 | 0 | 0 | 0 | 0 |
|  | 25-50% | 0 | 0 | 0 | 0 | 0 | 0 | 0 | 0 | 0 | 0 | 0 |
|  | 50-75% | 0 | 0 | 0 | 0 | 0 | 0 | 0 | 0 | 0 | 0 | 0 |
|  | 75-100% | 0 | 0 | 0 | 0 | 0 | 0 | 0 | 0 | 0 | 0 | 0 |
|  | >100% | 0 | 0 | 0 | 0 | 0 | 0 | 0 | 0 | 0 | 0 | 0 |
